# Blue-light induced biosynthesis of ROS contributes to the signaling mechanism of *Arabidopsis* cryptochrome

**DOI:** 10.1038/s41598-017-13832-z

**Published:** 2017-10-24

**Authors:** Mohamed El-Esawi, Louis-David Arthaut, Nathalie Jourdan, Alain d’Harlingue, Justin Link, Carlos F. Martino, Margaret Ahmad

**Affiliations:** 1 UMR CNRS 8256 (B2A), IBPS, Université Paris VI, Paris, 75005 France; 2 Botany Department, Faculty of Science, Tanta University, 31527 Tanta, Egypt; 3 Department of Physics, Xavier University, Cincinnati, Ohio, 45207 USA; 4 Department of Biomedical Engineering, Florida Institute of Technology, Melbourne, FL 32901 USA

## Abstract

Cryptochromes are evolutionarily conserved blue light receptors with many roles throughout plant growth and development. They undergo conformational changes in response to light enabling interaction with multiple downstream signaling partners. Recently, it has been shown that cryptochromes also synthesize reactive oxygen species (ROS) in response to light, suggesting the possibility of an alternate signaling mechanism. Here we show by fluorescence imaging and microscopy that H_2_0_2_ and ROS accumulate in the plant nucleus after cryptochrome activation. They induce ROS-regulated transcripts including for genes implicated in pathogen defense, biotic and abiotic stress. Mutant cryptochrome alleles that are non-functional in photomorphogenesis retain the capacity to induce ROS-responsive phenotypes. We conclude that nuclear biosynthesis of ROS by cryptochromes represents a new signaling paradigm that complements currently known mechanisms. This may lead to novel applications using blue light induced oxidative bursts to prime crop plants against the deleterious effects of environmental stresses and toxins.

## Introduction

Cryptochromes are blue light sensing receptors that regulate multiple processes of plant growth and development, including photomorphogenesis, de-etiolation, flowering initiation, stress response, and hormone signaling^1–3^. They are localized in the nucleus and function both directly and indirectly as core regulators of more than 20% of total cellular transcripts^4–6^. Structurally, cryptochromes are flavoproteins consisting of a conserved N-terminal light sensing domain (designated the PHR domain) and a less well-conserved C-terminal domain (designated CCE) of variable length that is important for nuclear localization and signaling^7^. Upon illumination, *Arabidopsis* cryptochromes interact with protein partners including phytochromes (PhyA and PhyB), the WD-repeat protein SPA1, the E3 ubiquitin ligase COP1, and bHLH transcription factors CIB^1,2^, PIF3 and PIF4^8,9^ which have a signaling role in photomorphogenesis or flowering initiation. Most of these partners attach to the PHR domain and release the CCE domain from the protein surface^10,11^. The ensuing suppression of COP1/SPA1-regulated degradation of transcription factors, such as HFR1 and HY5/HYH, results in photomorphogenesis and interaction with a large network of phytochrome, hormone, and stress signaling pathways^12–15^.

Recently, it has been reported that plant cryptochromes release reactive oxygen species (ROS) including hydrogen peroxide (H_2_O_2_) and superoxide (O_2_^•^−) subsequent to illumination^16,17^. This follows from reoxidation of the bound flavin cofactor from the light activated radical (FADH°) or reduced (FADH-) redox state back to the oxidized (FADox) resting dark-adapted state^18^. ROS are damaging byproducts of metabolism originating in the chloroplasts, peroxisomes or mitochondria. They are also induced by several environmental stresses and function as core regulators of cellular processes including stress tolerance, root growth, senescence, hormonal responses, pathogen defense, defense mechanisms against ROS and induction of ROS scavenging pathways^19–22^.

Intriguingly, cryptochromes have been indirectly implicated in a variety of ROS signaling pathways including defensive responses to singlet oxygen, high light stress, and apoptosis^23,24^. There is furthermore considerable overlap in genes regulated by ROS and by cryptochromes in microarray studies^24–27^. Some of these effects result from indirect regulation by cryptochrome of cellular downstream intermediates that alter the levels of production of singlet oxygen in response to light within the plastid^23^, or through regulation of known signaling intermediates involved in photomorphogenesis^26^. However, the mechanism of such cross talk remains poorly understood^24–27^. Given that cryptochrome illumination leads to the stoichiometric biosynthesis of ROS *in vitro*, address the possibility that direct biosynthesis of ROS by cryptochromes may indeed play a signaling role in response to blue light exposure in living plants.

## Results

**Figure 1:**
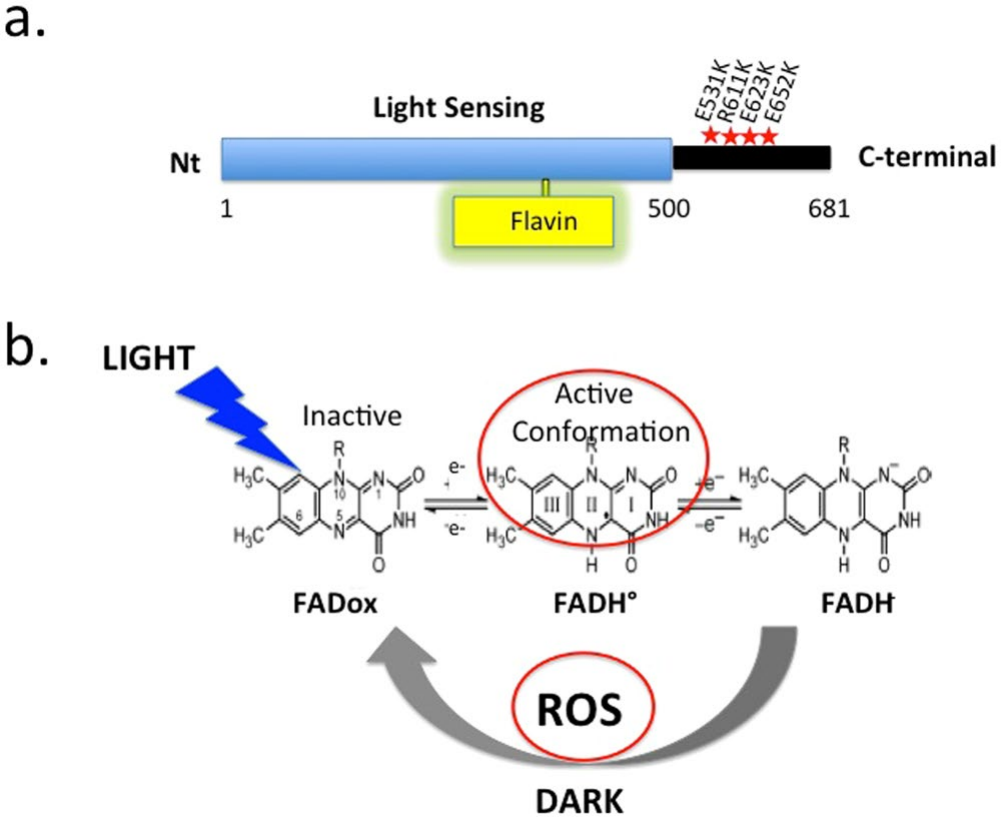
Domain structure and photochemical reactions of Arabidopsis cryptochrome 1. Upper panel light sensing (flavin binding) domain and C-terminal domain are indicated showing position of point mutants used in this study. Lower panel: flavin photoreduction and reoxidation reactions which have been linked to biological signaling. Flavin (FAD) is oxidized in the dark-adapted state of the receptor and becomes reduced in the presence of light, forming the neutral radical (FADH°) flavin redox state which is correlated with protein conformational change linked to biological activation. Subsequent to flavin reduction, flavin reoxidation occurs in a light-independent reaction leading to the production of Reactive Oxygen Species (ROS). Currently known paradigms for plant cryptochrome signaling are discussed in ref.^1^.

**Figure 2:**
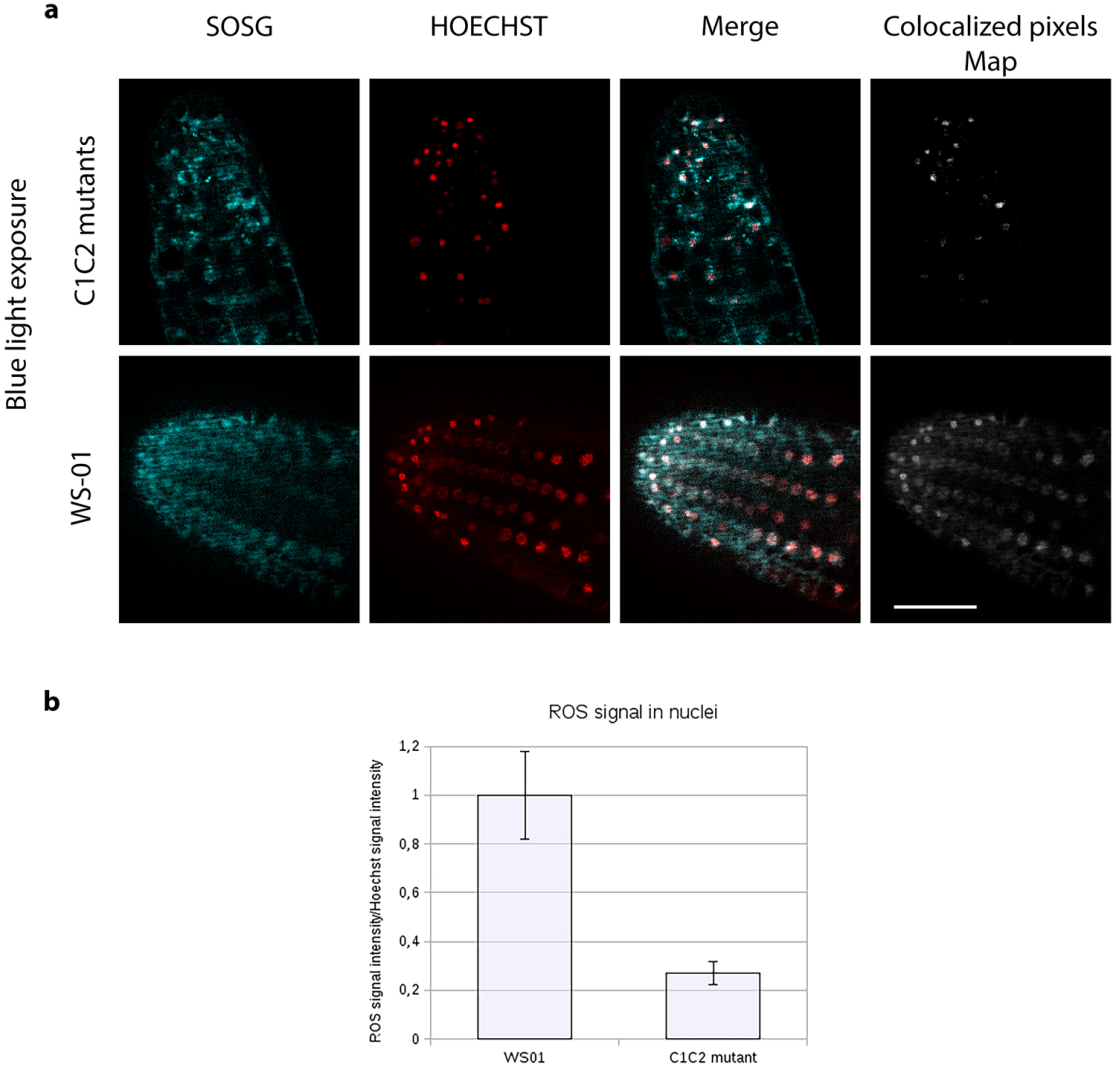
Production and subcellular localization of ROS in Cryptochrome-1 overexpressing (WS01) and double mutant *cry1cry2* (C1C2) seedlings exposed to blue light. Primary root tips from 8-day old etiolated Arabidopsis seedling (see methods) roots either lacking both cry1 and cry2 (C1C2 mutant) or over- expressing cryptochrome 1 (WS01) were treated with HOECHST for nuclear staining and SOSG for ROS staining for 30 minutes, exposed to blue light for 10 minutes, and then immediately viewed by a Zeiss AxioImager.Z1/ApoTome microscope. (**a**) Images show single z section that cross the nucleus. Colocalized pixels appear in white on the merged images and on the colocalized pixels maps. Scale bars are 25 μm. (**b**) using the segmentation and ROI manager tool on imageJ, fluorescence intensity of white and red pixels were quantified from these images for each nucleus and exported to LibreOffice calculator. The mean fluorescence intensity of >50 individual nuclei is shown.

Prior studies have shown that an increase in levels of ROS (reactive oxygen species) can be observed within minutes of blue light illumination in the nuclei of Sf21 insect cell cultures expressing recombinant *Arabidopsis* cryptochromes^16,17^. However, these observations have not  been linked to any physiological role in plants. We therefore first determined whether illumination would result in a detectable increase in levels of ROS in the nuclei of *Arabidopsis* plant seedlings. Cry – deficient mutant (*cry1cry2*) and cryptochrome overexpressing (Ws01) lines of the *Wassilewskija* (Ws) ecotype described previously^28^ were illuminated in the presence of the fluorescent probe Singlet Oxygen Sensor Green (SOSG) to detect singlet oxygen^26^, a byproduct of flavin reoxidation after illumination of cryptochromes (Fig. 1b)^18^. The apex of the primary root from 8 day old etiolated seedlings was assayed in order to avoid contribution of ROS from the chloroplasts (Fig. 2). After illumination, both cry-deficient (*cry1cry2*) and overexpressing (*WsO1*) Arabidopsis seedlings showed approximately similar levels of ROS, although levels were somewhat reduced in *cry1cry2* mutants (Fig. 2, SOSG panel). However, when fluorescence staining resulting from SOSG was overlapped with Hoechst stain for localization of the nuclei (see Merge, Fig. 2), a clear distinction between mutant and cry overexpressing lines could be observed. Quantification of fluorescent signal (SOSG) from these images indeed showed that colocalized with the nucleus was higher by almost 80% in the Ws01 cryptochrome overexpressing cells as compared to cry-deficient *cry1cry2* mutant seedlings (Fig. 2, Panel b). We conclude that increased ROS biosynthesis within the nuclear compartment occurs as a direct result of cryptochrome activation, consistent with a possible signaling role in the nucleus.

**Figure 3:**
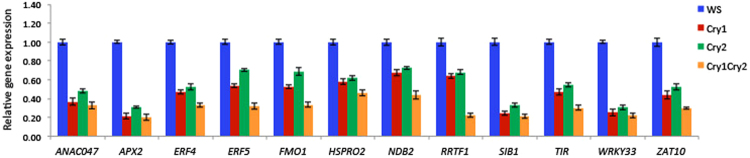
Effect of cryptochrome on transcription of representative ROS-regulated genes. Quantitative real-time PCR indicating the relative expression of twelve ROS-responsive genes investigated in WS (wild type), *cry1*, * cry2*, and *cry1 cry2* mutant seedlings. All seedlings were null mutants (absence of protein) ^28^. Four-day-old dark-grown seedlings were transferred to blue light (60 μmol m^−2^ s^−1^) for 3 h. prior to RNA extraction and qPCR analysis. Three replicates (biological) were used to compute bar errors (representing the S.D.).

Numerous transcriptome analyses of both cryptochrome-regulated and ROS regulated gene expression are available in several plant species and involving many different stress and illumination protocols^23–27^; reviewed also in refs^2,20^. These indicate considerable overlap between light-regulated and ROS regulated gene expression. To verify the role of cryptochromes in a variety of ROS signaling pathways, we chose to analyse at random a number of genes reported to respond to ROS across several distinct functional categories implicated in economically important traits such as defense against pathogen attack, and other forms of biotic and abiotic stress. These include ROS induced ethylene responsive transcription factors (ERF4,5)^29^ and WRKY33^27,^ the ROS regulated Zat zinc finger protein ZAT10^30^, Sigma Factor Binding Protein 1 (SIB1)^29^, Heat shock protein (HSPRO2)^27^, ROS detoxifying enzyme (APX2)^30^, oxidative stress responsive (ANACO47)^31^ and pathogen resistance gene FMO1^32^. The expression of these genes was analysed in *Arabidopsis* wild type (Wt) and mutant (*cry1, cry2, cry1cry2*) seedlings after a short (3 hour) illumination with saturating blue light to induce the cryptochrome response^33^ (Fig. 3). All of these genes showed reduced expression in cryptochrome-deficient mutant seedlings after illumination with blue light. No changes in expression were observed in the dark (Supplementary Fig. 1). Interestingly, expression is lower in either *cry1* and *cry2* single mutant seedlings as well, indicating that both proteins contribute towards a threshold of signal required for the transcriptional response.

**Figure 4:**
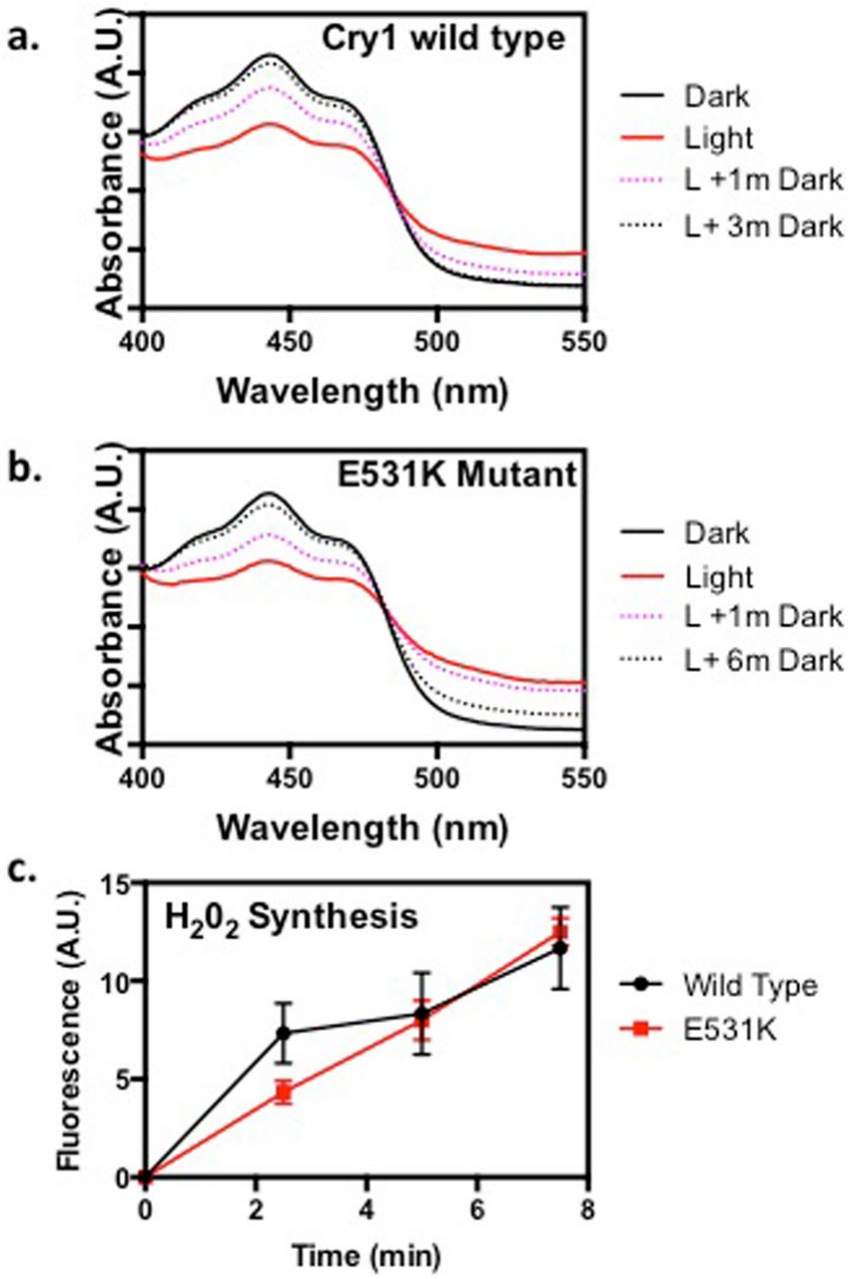
C-terminal point mutant of *Arabidopsis* cry1 retains light response and ROS biosynthetic capacity. Wild type (**a**) and E531K (**b**) mutant proteins were expressed and purified from baculovirus expression system as previously described^33^. ‘Dark’ shows spectra of isolated proteins prior to illumination. ‘Light’ indicates spectra after photoreduction for 30 seconds at 500 μmol m^−2^ s^−1^ blue light in the presence of 5 mM DTT . Samples were subsequently returned to dark and spectra taken at the indicated times (in min). (**c**). 100micromolar concentration of proteins were illuminated at 500 μmol m^−2^ s^−1^ blue light and aliquots taken at the indicated time for detection of ROS by AMPLEX RED fluorescence detection method as described^16^. Aliquots were taken at the indicated times. Error bars represent SD of three independent measurements.

Cryptochromes have been shown to undergo structural change in response to light, which allow their interaction with a number of downstream signaling partners leading to biological function^1,2^. This light-induced conformational change is essential for photomorphogenesis^2,12–15^ and appears also to contribute to the role of cryptochrome in  ROS signaling^23–26^. To resolve the question of whether direct biosynthesis of ROS by cryptochromes may by itself play a signaling role independently of conformational change, we analysed the biological activity of mutant alleles of CRY1 which had amino acid substitutions at their C-terminal (CCE) domain. Several such mutants had been previously described as inactive in hypocotyl growth inhibition and anthocyanin accumulation^33–35^, and as such can be concluded to be unable to undergo conformational change and partner protein interactions necessary for photomorphogenesis. To confirm that mutation of the C-terminal domain does not also affect light sensitivity or the formation of reactive oxygen (ROS) by the PHR domain of cryptochrome, we analysed protein from a mutant allele E531K^34^ which has a point mutation in the CCE domain outside of the region required for nuclear localization^36^. The recombinant E531K mutant protein was expressed and purified from a baculovirus expression system by established methods^33^, and compared to wild type AtCry1 for *in vitro* light sensitivity and flavin photoreduction. The mutant protein was comparable to the wild type AtCry1 both in the ability to undergo flavin photoreduction and the time course of reoxidation  from the radical (FADH°) state back to the oxidized (FAD) inactive redox state (Fig. 4a,b). We further tested the ability of the mutant protein to synthesise H_2_0_2_ in response to light, which was observed to be comparable to wild type (Fig. 4c). These data indicate that mutation within the C-terminal domain does not significantly affect cryptochrome photochemistry or the ability to synthesise ROS, and that reported loss of function in photomorphogenesis^34^ is most likely due to inability to undergo light dependent conformational change.

**Figure 5:**
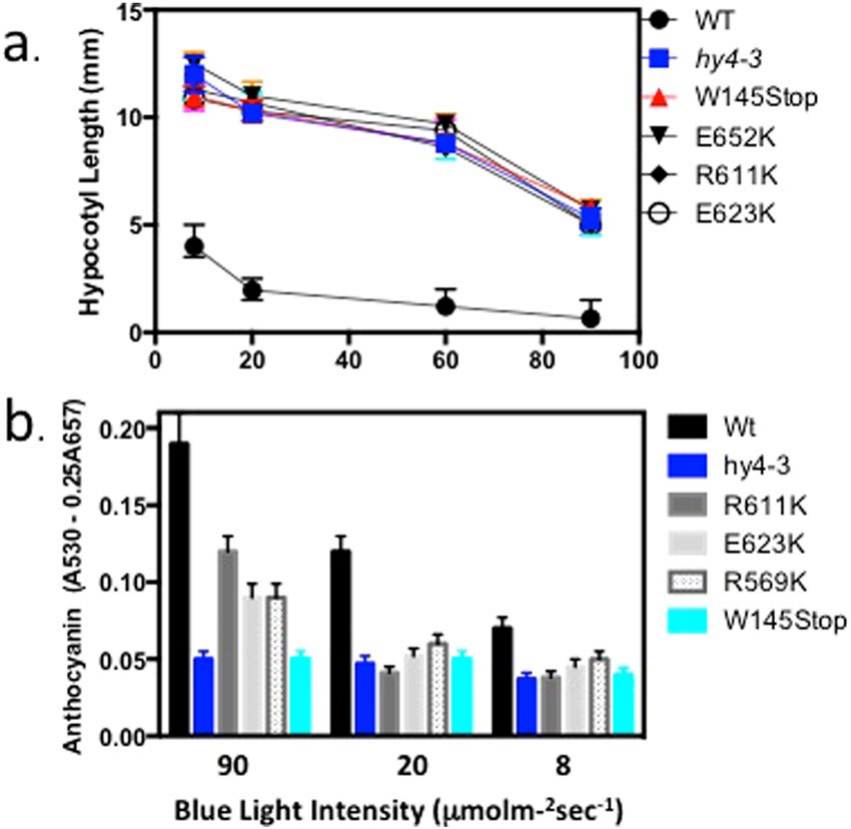
Phenotypes of cryptochrome C-terminal point mutants. Wild type and mutant alleles were grown for seven days on petri plates at the indicated light fluence (upper panel) and hypocotyl length of seedlings was measured. Error bars represent SD of measurement from 10 seedlings. Anthocyanin accumulation was determined as described^48^, error represents SD of three independent measurements.

To determine whether these mutant alleles retain the ability to mediate ROS signaling, we analysed the effect of amino acid substitutions in the C-terminal domain of CRY1 on responsivity to stress and ROS regulated gene expression. We chose only C-terminal point mutant alleles of AtCry1 for analysis (R611K, E623K and E652K) which were located outside of any known functional region for signaling *in vivo*, including the nuclear localization region^36^ and residues of the C-terminal implicated in phosphorylation^37^. These alleles all showed levels of protein expression similar to wild type (Supplementary Fig. 2) For comparison purposes, a null mutant allele lacking detectable protein (W145 – STOP) was used as a control in addition to the *cry1* reference allele (*hy4-3*), which also shows no detectable protein expression^28^. We first analysed these mutant alleles for hypocotyl growth inhibition at a range of blue light intensities (Fig. 5a, upper panel). All mutant alleles showed impairment in hypocotyl growth inhibition at blue light intensities ranging from 2 to 90 μmol m^−2^ s^−1^. These results indicate that all CRY C-terminal mutant alleles were biologically inactive for photomorphogenesis in spite of the fact that mutations in the C-terminal domain do not impede the capacity to synthesise ROS (Fig. 4). These results are consistent with a role for the C-terminal domain in inducing conformational change and interaction with signaling partners for these responses.

Anthocyanin accumulation is a highly regulated process occuring at the interface of numerous plant signaling pathways including photomorphogenesis. It is a consequence of both phytochromeand cryptochrome action. It is controlled by many of the same signaling intermediates that are implicated in other aspects of photomoprhogenesis such hypocotyl growth inhibition, such as HY5/HLH and COP1^38^. However, anthocyanin accumulation also occurs more specifically in response to multiple forms of stress, and we therefore investigated whether C-terminal mutants of Cry1 which continue to synthesize ROS may also retain some capacity to regulate this response (Fig. 5b). Indeed, the levels of anthocyanin were substantially reduced in all *cry1* mutant alleles in response to moderate intensities of blue light (at either 8 or 20 μmol m^−2^ s^−1^), consistent with previous reports^34,35^. However, at higher blue light intensity (90 μmol m^−2^ s^−1^), anthocyanin accumulation in the C-terminal mutant alleles R611K, E623K and E652K was significantly enhanced as compared to both of the null mutant alleles (Fig. 5b). These effects were not due to any overall partial activity (leakiness) of these C-terminal mutants, since there is no corresponding differential response in inhibition of hypocotyl elongation at any of these blue light intensities (Fig. 5a). These results indicate that cry1 mutants retaining the ability to synthesise ROS are also partially active in mediating anthocyanin accumulation under conditions of elevated stress (high light).

**Figure 6:**
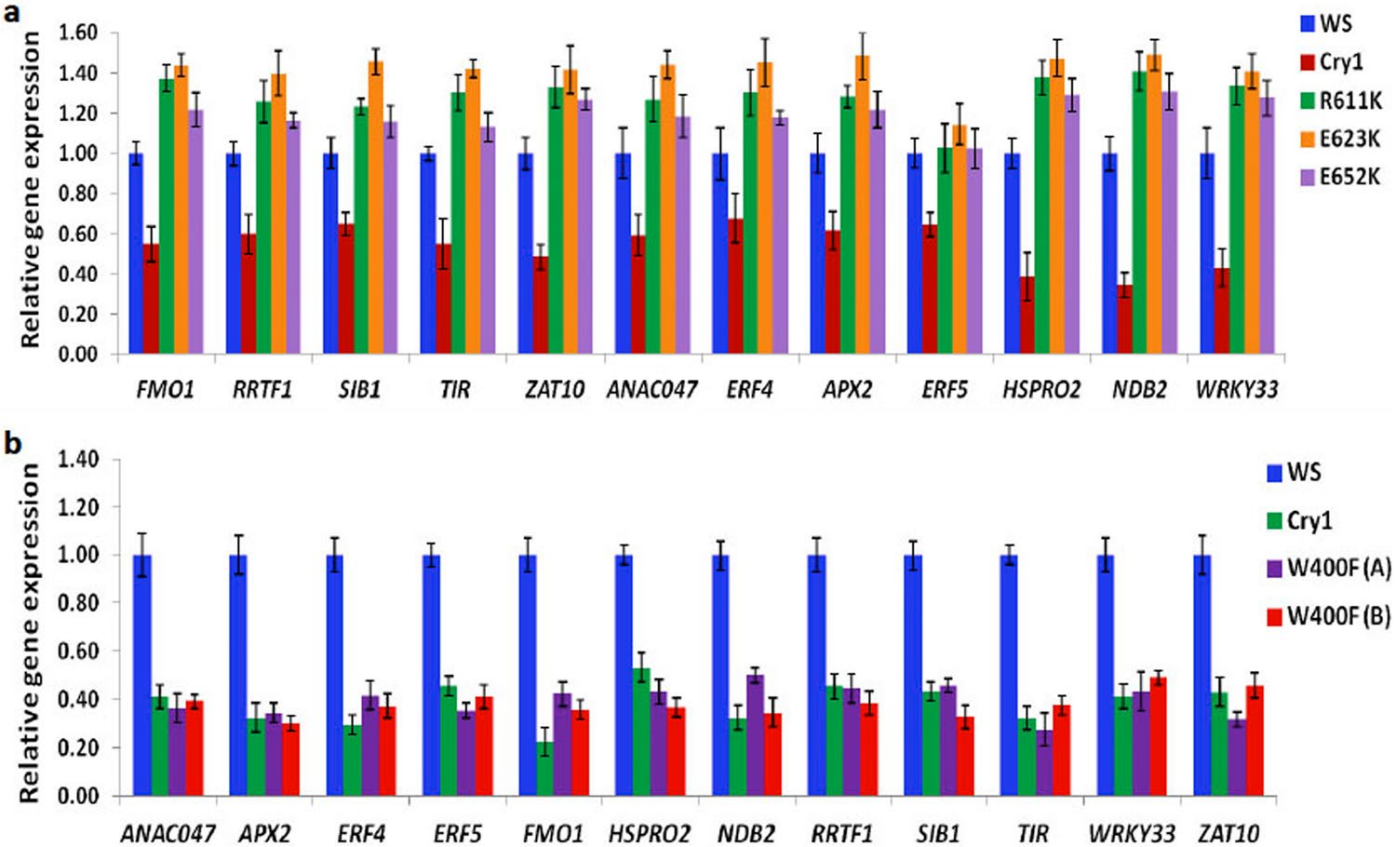
(**a**) C-terminal point mutants retain ability to induce ROS regulated gene expression. qPCR showing the relative expression of 12 ROS-responsive genes investigated in WS (wild type), Cry1 (null allele), and the indicated C-terminal point mutant seedlings. Four-day-old dark-grown seedlings were transferred to blue light (60 μmol m^−2^ s^−1^) for 3 h. prior to RNA extraction and qPCR analysis. Data are from three biological replicates *n* = 3; error bars indicate ± SD. No differences in expression were noted in dark grown seedlings without blue light treatment (supplementary Fig. 1). (**b**) Expression of ROS regulated genes in Trp triad W400F mutant Arabidopsis seedlings. qPCR shows expression of 12 ROS regulated genes in wild type (Ws), cry1 mutant, and expressing W400F Trp triad mutants (lines A and B - see Supplementary Fig. 3 for levels of mutant protein expression). No effect on gene expression is observed for the control cry mutant alleles. Data are from 3 biological replicates (n = 3), error bars indicate SD.

Finally, we analysed the C-terminal point mutant alleles for their ability to induce ROS regulated gene expression characterized in Fig. 3, as all of these mutants are flavin bound and synthesize ROS as isolated proteins *in vitro* (Fig. 4, see also Supplementary Fig. 3). As can be seen, all three C-terminal point mutant alleles R611K, E623K, and E652K induced the expression of ROS regulated genes in response to blue light (Fig. 6a). This was the case moreover for each of the twelve chosen target genes, which have been implicated in several distinct and independent ROS-regulated response pathways (see Fig. 3). Interestingly, levels of induced gene expression were in some cases even somewhat enhanced in these Cry mutant alleles as compared to wild type, although levels of Cry protein expression in all of the mutants was the same (Supplementary Fig. 2). This may have been due to mechanisms of  homeostasis via conformational change in which the C-terminal mutants were deficient. Taken together with the results for anthocyanin accumulation (Fig. 5), these data show that C-terminal mutants of cryptochrome which have lost the ability to undergo conformational change nonetheless retain ROS signaling function.

## Discussion

In this work, we have addressed the important question of how cryptochromes interact with ROS signaling pathways in response to oxidative stress. We show that there is a visible accumulation of ROS in Arabidopsis seedlings as a result of cryptochrome activation by light (Fig. 2). This occurs also in the nucleus, and coincides with the rapid induction of ROS-regulated genes implicated in a number of different cellular response pathways (Fig. 3). Significantly, cryptochrome C-terminal mutant alleles that are impaired in normal light regulated conformational change and photomorphogenesis still retain the ability to synthesize ROS (Fig. 4 – See also Supplementary Fig. 3). These mutant alleles likewise retain partial activity in the regulation of stress response such as anthocyanin accumulation (Fig. 5). They furthermore retain an active signaling role in the induction of ROS-responsive genes (Fig. 6a). We note that seedlings expressing a mutation in the Trp triad residue W400F, which shows partial deficiency of flavin photoreduction *in vivo* ^1^, and thereby deficiency in biosynthesis of ROS, did not induce ROS responsive genes (Supplementary Fig. 2, Fig. 6b). Furthermore, seedlings overexpressing solely the N-terminal flavin binding domain of CRY1 reportedly induced many of the ROS responsive genes characterized in our study^37^. All of these data are therefore consistent with light dependent biosynthesis of ROS by cryptochrome as a possible signaling mechanism in the cases we have documented. Further transcriptome analysis will be required in order to clarify the number of cellular signaling pathways and the extent to which this mechanism may hold true.

The ROS signaling effects documented here in the C-terminal Cry1 mutant alleles appear to operate by distinct mechanisms than those described in previous reports of response to ROS in which cryptochromes have been implicated^23,26^. The previous studies show cryptochrome are implicated in ROS signaling pathways by indirect mechanisms involving regulation of singlet oxygen production by the chloroplast^23^ or by interaction with COP1 and transcription factors such as HY5/HYH which act downstream of cryptochromes in photomorphogenesis and plant growth. These processes require conformational change by cryptochromes which are lacking in C-terminal mutants of Cry1 (Fig. 4a). Therefore, blue-light induced signaling by C-terminal mutants likely involves direct synthesis of ROS and must function additively with, and act in a complementary manner, with the known signaling mechanism involving blue-light dependent conformational change.

Despite their capacity to synthesize ROS, cryptochromes would not be predicted to have a great impact on overall ROS biosynthesis in the cell; certainly nothing comparable to other sources of ROS such as in the plastid and mitochondria. This is because the cryptochrome photocycle is relatively slow, with a half-life of the reduced flavin state of several minutes^38^. Therefore, over several minutes, only a few molecules of ROS would be synthesized per cryptochrome even under saturating blue light conditions. Nonetheless, a unique feature of cryptochromes that is not present in other generators of ROS is their nuclear localization and potential proximity to redox sensitive signaling intermediates. This can greatly increase the effective concentration of ROS induced by blue light, as the most likely targets of ROS synthesised by cryptochromes may be transcription factors or other signaling intermediates to which they are in close proximity^22^.

One likely substrate, would be the family of G-Box binding transcription factors including GBF1 and MYC2, which are known to bind promoter elements related to high light stress signaling^39^. G-box binding factors are negative regulators of transcription whose binding affinity to DNA is shown to be greatly impaired by the presence of H_2_0_2_, resulting in their release from these promoters and subsequent induction of transcription^40^. G-box binding transcription factors are localized in close proximity to cryptochrome in the nucleus, as both are bound either directly or indirectly to the core regulator COP1 (E3 ubiquitin ligase)^2,39^. Since cryptochromes bind to COP1 independently of light, they would be in permanent close proximity to the G-box binding family of transcription factors, which bind to COP1 through the intermediary of HY5/HLH transcription factors^39,40^. Therefore, a pulse of blue light illumination would provide a localized burst of H_2_0_2_ sufficient to induce a transcriptional response from GBF-regulated promoters. Along these lines, biosynthesis of ROS by cryptochromes may  contribute to its signaling role simply by increasing the local concentration of ROS near transcription factors to which cryptochromes have not been reported to bind directly, but which are assembled into the same complexes. Recent studies indicating a multiplicity of signaling molecules that are regulated directly by oxidation/reduction at cysteine residues greatly adds to the potential signaling substrates of cryptochromes^41^.

During photomorphogenesis, the predominant cryptochrome signaling mechanism is that involving conformational change and interaction with downstream signaling partners such as COP1 and SPA1. This is evident from the fact that mutants at the C-terminal domain completely abolish photomorphogenesis under normal plant growth conditions even though they can still synthesize ROS (see Fig. 4). This is consistent with the fact that, under steady state conditions, ROS levels are not primarily determined by environmental conditions (such as high light intensity) but rather by the interplay between the ROS producing and ROS-scavenging pathways, leading to a tightly regulate redox state equilibrium^30^. Given this process of redox homeostasis, induction of ROS through cryptochrome activation would not be expected to change intracellular levels of ROS under steady state, long-term growth conditions. Furthermore, under subsaturating light conditions, the efficacy of ROS formation as a signaling mechanism is doubtful as the quantum yield of the receptor is relatively low^33^, so that a mechanism of conformational change, wherein the activated form of cryptochrome is stable over a period of at least several minutes^38^ is the optimal signaling mechanism. As a result direct induction of ROS signaling pathways through cryptochrome is likely to occur primarily under short-term illumination at high light intensities, both to ensure saturating light intensities for cryptochrome-dependent ROS biosynthesis and to evade eventual cellular ROS scavenging and downregulation mechanisms. As a validation of this prediction, we observe that under the short term, high intensity illumination conditions used in this study we have observed robust cryptochrome-dependent induction of the ROS regulated FMO1 gene (involved in pathogen defense – Figs 3 and 6), whereas in prior studies carried out under continuous illumination in white light with this same gene there was no reported evidence for cryptochrome control^32^. Along these lines, transcriptome analyses of cryptochrome dependent gene expression under high light shows preponderance of stress regulated transcripts in Arabidopsis^24^, whereas different illumination conditions have not shown significant up-regulation of ROS responsive genes as a result of cryptochrome activation in Rhodobacter^42^ or in Brassica^43^, for example, although the converse appear to be true for tomato^44^.

From a practical perspective,  almost all environmental stresses leading to economic loss such as extremes of temperature, salinity, drought, heavy metals, herbicides and pathogens involve formation of ROS, with concommittant induction of plant defense and protective mechanisms. The increase in ROS are generally transient and occur at the onset of the stress. Intriguingly, a number of studies have shown that pre-exposure to subthreshold levels of an “oxidative burst” can actively trigger a protective function or immunize plants against subsequent environmental stresses, a process known as acclamatory stress tolerance^45–47^. Cryptochromes are therefore ideal targets for such interventions. They can be completely photoreduced within less than a minute of illumination at light intensities which can be regulated to achieve desired levels of ROS directly within the nuclear compartment . Thus, a series of appropriately spaced blue light pulses can be delivered in the course of a night break which are sub-threshold in terms of inducing any form of oxidative damage or interrupting plant sleep/wake cycles, but sufficiently strong to induce desired plant defense mechanisms against selected environmental stress.  Manipulation of cryptochrome responses may thereby become a new optogenetic tool to improve crop plant productivity at minimal cost and without chemical intervention.

## Materials and Methods

### Seedlings and plant growth conditions

All Arabidopsis seedlings were of ecotype WS. Cry1 (*hy4-3*) and cry2 mutant reference alleles^28^ lacked detectable protein. Other mutant alleles were either as previously described^34,35^ or screened and sequenced from genetic screens for blue light insensitive mutants as in ref.^35^. All seedling growth was on agar petri plates at 1/2xMS salts, 2%sucrose, pH 6.0, 0.8% agar. Seeds were sterilized as in ref.^28^, plated, retained at 4 °C for two days, then allowed to germinate for 24 hours in red light before being returned to darkness or the test light condition. Hypocotyl growth and anthocyanin content was measured as previously described^35^.

### Imaging Experiments

10 plantlets (8–10 days old etiolated seedlings) were used per experiment and incubated in 2 ml of 200 mM potassium phosphate buffer (pH 7.4) comprising 2 µL HOECHST reagent (10 µM, Thermo Scientific), 2 µL DMSO (5 µM, Sigma -Aldrich) and 4 µL of SOSG (10 µM, Molecular Probes) for 30 minutes in the dark at room temperature. Plantlets were then rinsed 4 times in potassium phosphate buffer (200 mM), then exposed either to dark or to saturating blue light (900 µm m^−2^ s^−1^) for 10 minutes and observed immediately with a Zeiss AxioImager.Z1/ApoTome microscope using a 20X objective, excitation/emission 350 nm/460–490 for HOECHST and 372/395–416 for SOSG reagent. Z- series were performed with a z-step of 1.5 µm. For quantification, the images were captured using a CCD-camera. Single z section were analysed for colocalization on ImageJ software (W. S. Rasband, ImageJ). Briefly, colocalized pixels were obtained by using colocalization color map plugging. Red structures on Hoechst images have been segmented to define each nucleus as a region of interest (ROI), then by using ROI manager tool, integrated density of cyan and red signal have been quantified for each ROI and exported to LibreOffice calculator.

### Quantitative RT-PCR analysis

The seedlings of *Arabidopsis thaliana* wild-type (WS), *CRY1* null mutant, *CRY2* mutant, *CRY1-CRY2* double mutant and C-terminal mutants of *Arg611Lys*, *Glu623Lys* and *Glu652Lys* were grown and germinated as described above. Four-day-old dark-grown seedlings were transferred to blue light (60 μmol m^−2^ s^−1^) for 3 h or kept in dark. Seedlings were then collected and ground with liquid nitrogen. Total RNA was isolated using RNeasy Plant kit (Qiagen), and the first-strand cDNA synthesis was done using a Reverse Transcription Kit (Qiagen) following the manufacturer’s instructions. qRT-PCR was performed using a SYBR Green PCR kit (Qiagen) according to the manufacturer’s instructions. All qRT-PCR reactions were amplified as follows: 95 °C for 15 min, followed by 40 cycles of 95 °C for 15 s, 57 °C for 30 s, and 72 °C for 30 s. The expression levels were determined and normalized to the *UBIQUITIN* (*UBQ1*) expression using the comparative 2^−∆∆Ct^ method. Primers of the 12 ROS genes used for gene expression analysis are listed in Supplementary Table 1.

### Protein Expression, extraction, and ROS detection

All methods were as previously described^16^. Briefly, the mutant allele E531K of cry1 was cloned into the pBakPak9 baculovirus expression vector (Clontech, inc.)including the presence of a 6 amino acid HIS tag at the N-terminus. The vector was transfected into Sf21 insect cell cultures and protein expression carried out according to the manufacturer’s instructions. Recombinant protein was isolated over a nickel affinity column as described^48^. Concentration of protein used in the experiments was between 30–50 micromolar. Photoreduction experiments were peformed in a buffer of 50 mM NaPO_4_ pH 7.5, 5 mM DTT at18 °C. Subsequent to illumination, spectra were taken in a Cary Scan 300 UV/Vis spectrophotometer. For detection of ROS, samples of wild type Cry1 and E531K mutant proteins at the identical concentration were illuminated in a buffer of 50 mM NaPO_4_ pH 7.5 maintained on ice. Ilumination was at 1000 μmol m^−2^ s^−1^ in the absence of reducing agents. Aliqots were taken at the indicated times (Fig. 4) and frozen in liquid nitrogen. At the conclusion of the time course, samples were thawed and aliquots tested using the Amplex Red fluorescence detection system (Molecular Bioprobes)  to determine the concentration of H_2_0_2_ as described^16^).

## Electronic supplementary material

Supplementary Information
