# Supplementary material for: Blue-light induced biosynthesis of ROS contributes to the signaling mechanism of Arabidopsis cryptochrome
Source: Sci Rep. 2017 Oct 24;7:13875. doi: 10.1038/s41598-017-13832-z (PMC5655019; doi:10.1038/s41598-017-13832-z)
Supplement: Supplementary file 1 — Supplementary Information [file 41598_2017_13832_MOESM1_ESM.docx]

**Supplementary Information.**

**Blue-light induced biosynthesis of ROS contributes to the signaling mechanism of *Arabidopsis* cryptochrome*.***

Mohamed El-Esawi ^1,2^, Louis-David Arthaut^1^, Nathalie Jourdan^1^, Alain d’Harlingue^1^, Justin Link^3^, Carlos F. Martino^4^ and Margaret Ahmad^1,3*^.

^1^ UMR CNRS 8256 (B2A), IBPS, Université Paris VI, Paris, 75005 France.

^2^ Botany Department, Faculty of Science, Tanta University, 31527 Tanta, Egypt.

^3^ Department of Physics, Xavier University, Cincinnati, Ohio, 45207, U.S.A.

^4^ Department of Biomedical Engineering, Florida Institute of Technology, Melbourne, FL 32901 U.S.A.


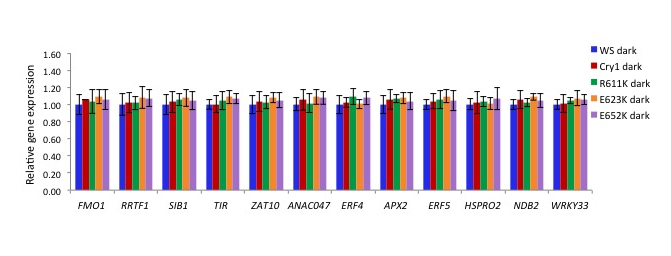


Supplementary Figure 1. Expression of ROS regulated genes in dark-grown *Arabidopsis* seedlings. Quantitative real-time PCR shows expression of 12 ROS regulated genes in wild type (Ws) and cry1 mutant alleles in 4-day old dark-grown seedlings. No effect on gene expression is observed for any of the cry mutant alleles. Data are from 3 biological replicates (n=3), error bars represent S.D.

**Supplementary Table 1. List of primers used in the current study**

1. **Primers of the ROS genes used for gene expression analysis (qRT-PCR)**

| **Gene** | **AGI** | **Primer name** | **Primer sequence (5'-3')** | **Primer Reference** |
| --- | --- | --- | --- | --- |
| *ANAC047* | At3g04070 | NAC-QF | ACCCCTTAAACGCCAGAAGT | Designed in this study |
|  |  | NAC-QR | ATGCCGCTTGTCTCTGAAGT |  |
| *APX2* | At3g09640 | APX2-QF | ACAAAGTTGAGCCACCTCCT | Chen *et al*. (2013)* |
|  |  | APX2-QR | AAGGTGTGTCCACCAGACAA |  |
| *ERF4* | At3g15210 | ERF4-QF | GGATCGGTAACGTAGGTCGT | Chen *et al*. (2013) |
|  |  | ERF4-QR | ATCCCACCTTCGAAATCAAC |  |
| *ERF5* | At5g47230 | ERF5-QF | CATCTTCGGATCATCGTCCT | Designed in this study |
|  |  | ERF5-QR | ATCTTCAATGGCGGTTTACG |  |
| *FMO1* | At1g19250 | FMO1-QF | CTTCTACTCTCCTCAGTGGCAAA | Designed in this study |
|  |  | FMO1-QR | CTAATGTCGTCCCATCTTCAAAC |  |
| *HSPRO2* | At2g40000 | HSPRO2-QF | CTCCGATCTCATGTTCAGCA | Designed in this study |
|  |  | HSPRO2-QR | TGGATCTGCTTCGTGATGAG |  |
| *NDB2* | At4g05020 | NDB2-QF | TATTCTGAGGCAAACGCATC | Chen *et al*. (2013) |
|  |  | NDB2-QR | AGCAGCACCACCTTCTTCTT |  |
| *RRTF1* | At4g34410 | RRTF1-QF | CACCAACAGAGTCGCAAGAA | Designed in this study |
|  |  | RRTF1-QR | ACTGGAAAAACCCTGACACG |  |
| *SIB1* | At3g56710 | SIB1-QF | CGTTCGGTGAGAGAGACAGT | Chen *et al*. (2013) |
|  |  | SIB1-QR | TCGATGCTTCCAAAGTCATT |  |
| *TIR* | At1g57630 | TIR-QF | GCTTTCACGGGGAAGATGTA | Designed in this study |
|  |  | TIR-QR | TGATCTCCACCAATTCGTCA |  |
| *WRKY33* | At2g38470 | WRKY-QF | GGGGACAATGAAACAAATGG | Designed in this study |
|  |  | WRKY-QR | TGTCGTGTGATGCTCTCTCC |  |
| *ZAT10* | At1g27730 | ZAT10-QF | ATCACACGTTTGCACCATCT | Chen *et al*. (2013) |
|  |  | ZAT10-QR | TGCTAACGTGGCTAGTGGAC |  |
| *UBQ1* | At3g52590 | UBQ-QF | TTCCTTGATGATGCTTGCTC | Chen *et al*. (2013) |
|  |  | UBQ-QR | TTGACAGCTCTTGGGTGAAG |  |

*Chen, D., Xu, G., Tang, W., Jing, Y., Ji, Q., Fei, Z. & Lin, R. Antagonistic Basic Helix-Loop-Helix/bZIP Transcription Factors Form Transcriptional Modules That Integrate Light and Reactive Oxygen Species Signaling in Arabidopsis. *The Plant Cell* **25,** 1657–1673 (2013).


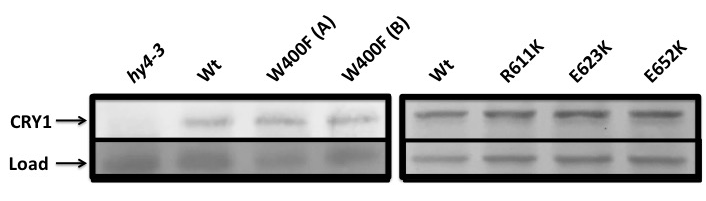


Supplementary Figure 2. Expression of mutant cryptochrome proteins in 4-day old *Arabidopsis* seedlings. Dark-grown Arabidopsis seedlings of the indicated genotypes were harvested into liquid nitrogen, homogenized and equivalent protein concentrations resolved on each lane of a 10% polyacrylamide gel prior to Western blot analysis using anti-CRY1 antibodies. The blots were then probed with non-specific HSP90 antibody as loading control. Left upper panel: Western blot signal resulting from anti-Cry1 antibody for cryptochrome mutant (*hy4-3*) and W400F seedlngs of two (A and B) independent transgenic lines. Left lower panel: Western blot signal of upper left panel blot probed with non-specific antibody to indicate equivalent protein load. Right upper panel: Western blot signal resulting from anti-Cry1 antibody for C-terminal Cry point mutants. Right lower panel: Western blot signal of upper right panel blot probed with non-specific antibody to indicate equivalent protein load. These blots show that expression of mutant cryptochrome proteins is comparable to wild type.


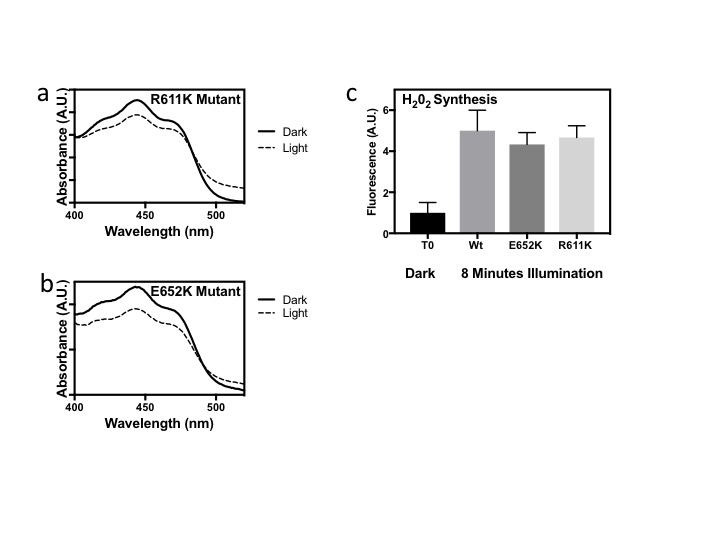


Supplementary Figure 3. Flavin binding and production of ROS by cry1 C-terminal mutants R611K and E652K. Additional mutant proteins were isolated from baculovirus expression system as described [48] – see also figure 4. a, b: Spectra were taken of purified samples before (Dark) or after (Light) 1 min illumination in blue light (450nm) at 500μmol m^-2^ s^-1^  in the absence of added reductant. Shift in absorption spectra indicate flavin is bound and photochemically active. Flavin reduction of wild type sample was comparable under this illumination condition (not shown). C. Production of ROS by isolated protein samples was determined as described in the text (see Fig.4, and methods) either before (Dark) and after 8 minutes of illumination at 500μmol m^-2^ s^-1^ . Error bar represents S.D. of three measurements.
